# Antioxidant and Antibacterial Activities of the Essential Oil of Moroccan *Tetraclinis articulata* (Vahl) Masters

**DOI:** 10.1155/2020/9638548

**Published:** 2020-06-25

**Authors:** Halima Rabib, Chaimaa Elagdi, Mohammed Hsaine, Hassan Fougrach, Tayeb Koussa, Wadi Badri

**Affiliations:** ^1^Laboratory of Plant Biotechnology, Ecology and Ecosystem Valorization, Department of Biology, Faculty of Sciences El Jadida, Route Ben Maachou, El Jadida 24000, Morocco; ^2^Laboratory of Ecology and Environment, Department of Biology, Faculty of Sciences Ben M'sik, P.B. Av Driss El Harti Sidi Othman, Casablanca 7955, Morocco

## Abstract

The purpose of this study is to evaluate and compare the antioxidant and antibacterial activities of essential oil isolated from *Tetraclinis articulata* (Vahl) leaves, Masters originating in Morocco (Benslimane Region, Atlantic-influenced plain). The analysis of the major compounds of essential oil was performed by gas chromatography and mass spectrometry, and this oil is dominated by bornyl acetate (35.05%), camphor (11.17%), and *α*-pinene (10.84%). The antioxidant properties were evaluated by the test of the radical trap 2,2-diphényl-1-picrylhydrazyl (DPPH), and the antimicrobial activity of *T*. *articulata* essential oil was tested against clinical isolates of *Staphylococcus aureus*, *Pseudomonas aeruginosa*, and *Escherichia coli* which have been inhibited from the 25 *μ*g/mL.

## 1. Introduction

As part of our work on the essential oils of *Tetraclinis articulata* [1], this plant is a very slow-growing coniferous species, longevity of which may exceed 400 years. In height, the annual increment is estimated to be 20 cm/year up to 25 years and 10 cm/year up to 60 years. In diameter, it is 3 to 4 mm/year up to 30 years and 1.5 to 2.5 mm/year up to 60 years. These numbers depend on several parameters, including soil depth and annual precipitation [2].

It is characterized by a clear crown and an irregular pyramid port, and its foliage is persistent; the leaves appear vertically by four, long welded to the stem. The twigs, green, flattened, and covered with articulated leaves, hence the name *T. articulata* [3].

The majority of the Tetraclinaie area of Morocco is located in the semiarid bioclimatic floor with a moderate, gentle, fiery variant. They can also grow in the subhumid with a warm, mild, or temperate variant on filterable soils, on dense slopes or on hot lights [4]. It has always been used in folk medicine due to its varied therapeutic effects; it is more used against intestinal and respiratory infections also against skin infections [5, 6].

The use of synthetic antioxidant molecules is currently being questioned due to potential toxicological risks. New plant sources of natural antioxidants are now being sought [7, 8]. Indeed, the polyphenols are natural compounds that are widespread in the plant reign, which are of increasing importance through their health benefits [9]. Their role as natural antioxidants is gaining interest in cancer prevention and treatment and inflammatory and cardiovascular diseases [10].

The antimicrobial properties of essential oils have been known and used for a long time, but this use was based on traditional practices [11] and applications without precise scientific bases. These days, they are used on scientific and rational grounds, since many research studies are conducted on the antimicrobial properties of EOs in aromatic plants [12–15].

In the literature, studies on the biological activities of *T. articulata* oils have shown that these species are antibacterial, antifungal, and cytotoxic [16–19].

This work is part of the research and development of bioactive substances such as natural substances with antioxidant activity of interest in biopharmacology. The main objective of this work is to assess *in vitro* the essential oil antioxidant activity of the aerial part of *Tetraclinis articulata* (Vahl) using the method of DPPH free radical trapping and the antibacterial activity on three strains: *Staphylococcus aureus*, *Pseudomonas aeruginosa,* and *Escherichia coli.*

## 2. Materials and Methods

### 2.1. Plant Material

The leaves of *T. articulata* were collected from Benslimane Region precisely in Ain Dakhla Atlantic-influenced plain (N 33 40.688 W 7 00.494, altitude: 207 m), and this region has a mediterranean climate and a limestone soil. The harvest was carried out on trees taken at random in January 2018. The identification of the plant was made in our laboratory. The samples were then dried in air at room temperature (25°C) and were protected from light for nine days.

### 2.2. Extraction of Essential Oil

Extraction of the essential oil from the aerial part of the tree was carried out by steaming with a Clevenger-type apparatus [20], in a 5-liter balloon containing 2 liters of distilled water. The extractions were repeated three times in order to recover considerable volumes. The essential oil obtained has been stored in small, opaque bottles and placed in a refrigerator at 4°C until used for testing antioxidant and antibacterial activities.

### 2.3. GC/MS Analysis

Three chromatographic analyses were performed, and the essential oil was analyzed on a Hewlett-Packard Gas Chromatograph Model 5890 coupled to a Hewlett-Packard Model 5971, equipped with a DB5 MS column (30 m × 0.25 mm; 0.25 *μ*m), programming from 50°C (5 min) to 300°C at 5°C/min, with a 5-min hold. The helium was used as the carrier gas (1.0 mL/min); injection was in split mode (1:30); injector and detector temperatures were 250°C and 280°C, respectively. The mass spectrometer worked in EI mode at 70 eV, electron multiplier at 2500 V, and ion source temperature at 180°C; MS data were acquired in the scan mode in the m/*z* range 33–450.

The majority constituents of essential oil were identified in comparison with their specters of mass with those of the NIST mass spectral library.

### 2.4. Antioxidant Activity

The antioxidant activity of a compound corresponds to its ability to resist oxidation. Indeed, most synthetic or natural antioxidants have the hydroxyphenolic group in their structures and the antioxidant properties are attributed in part, to the ability of these natural compounds to trap free radicals such as hydroxyl radicals (OH^•^) and superoxides (O_2_^*·*^) [21].

**Figure 1:**
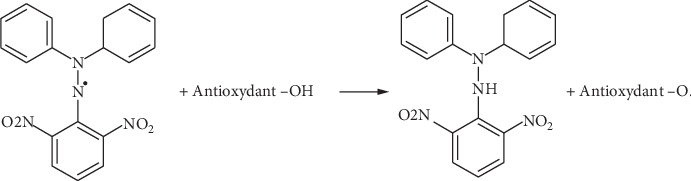
The reaction of an antioxidant with the radical DPPH.

The measurement of antiradical activity was tested using the Blois method [22] as described by Brand-Williams et al. [23] with some modifications. The principle of this method is based on the measurement of DPPH (2,2-diphenyl-1-picrylhydrazyl) free radical scavenging in solution in methanol according to the reaction in Figure 1. The spectrophotometer measurements are made at 517 nm. It, therefore, provides a practical means of measuring the antioxidant activity of essential oils.

Thus, from a standard oil solution of 2 mg/mL, daughter (diluted) solutions were prepared by successive dilution in methanol. Then, at each concentration, a volume of a methanol solution of DPPH was added. The reaction mixture was incubated in the dark at room temperature for 30 minutes. The vitamin C and butylhydroxytoluene (BHT) were prepared in the same condition and then used as a standard.

At the end of the incubation period, the absorbance at 517 nm is read and the antioxidant activity is calculated according to the following equation:

(1) $$\begin{array}{c} \%\text{IP}=\;\left\{\frac{\left(\text{Abs control}-\text{Abs sample}\right)}{\left(\text{Abs control}\right)}\right\}\times 100, \end{array}$$

where %IP is the percentage of inhibition and Abs is the absorbance at 517 nm.

### 2.5. Antibacterial Activity

The aromatogram was made on Petri dishes filled with Muller-Hinton agar. The agar is inoculated with 100 *μ*L of suspensions of 10^8^ cfu/mL and then deposited on its surface of Whatman paper discs (6 mm) impregnated with 10 *μ*L of the essential oil. In order for the essential oil to diffuse, Petri dishes are then closed and left at room temperature for 30 min, then incubated at 37°C for 24 h.

Once incubation is complete, the interpretation of the results is performed by measuring the inhibition zones [24].

The minimum inhibitory concentration (MIC) was evaluated according to published procedures [25, 26]; these concentrations were determined by the CLSI microdilution method (2006) on 96-well round bottomed microplates with some modifications. For a concentration of 10^5^ cfu/mL, 10^8^ cfu/mL bacterial suspensions were diluted 1/1000 with the same culture medium (TS).

Essential oil has been diluted by Tween 80 to 1% to have a concentration of 500 mg/mL, and then the first 10 columns of the microplate are filled by the different concentrations of essential oil; from this solution, a series of ½ fold dilutions were prepared to obtain a concentration range between 500 and 1 mg/mL. On microplates, the negative control (column N°11) was chosen to fill the wells with 100 *μ*L sterile broth. Positive control wells (column N°12) were filled with 100 *μ*L of the standardized microbial suspension at 10^5^ cfu/mL. Then, the plates were incubated at 37°C for 24 hours [1].

## 3. Results and Discussion

### 3.1. The Majority Component of Essential Oil

The average yield of essential oil extracted from the leaves of *T. articulata* (Vahl) is 0.5%. This oil, pale yellow, has a strong balsamic smell.

The essential oil contents of the fresh and dry leaves of *Tetraclinis articulata* from different Moroccan stations vary from 0.06 to 0.81% and are, therefore, very close to our value [17, 27–30].

**Figure 2:**
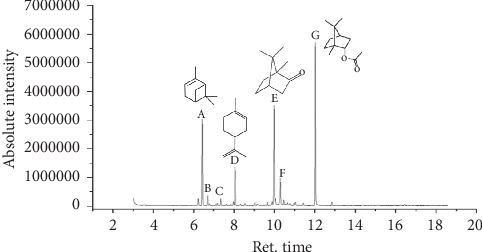
Chromatogram of the major components of *Tetraclinis articulata* essential oil ((A) *α*-pinene, (B) camphene, (C) *α*-thujone, (D) limonene, (E) camphor, (F) borneol, and (G) bornyl acetate).

The GC/MS analysis of essential oil identified seven majority constituents (Figure 2).

Our essential oil is rich in monoterpenic hydrocarbons (35.18%) and esters (35.05%); the following table represents the majority components with their percentage (Table 1).

The results of the chromatographic analysis show that this essential oil is very rich in monoterpenic hydrocarbons and the bornyl acetate (35.05%), and camphor (11.17%) and *α*-pinene (10.84%) are the clear majority compounds. A study by Bourkhiss et al. showed that essential oil from Essaouira is rich in camphor (31.6%) and the bornyl acetate (25.4%) [31].

Two compositions have been identified in the Khemisset region, and the majority compound of this oil is bornyl acetate (30.7% and 30.6%, respectively) followed by *α*-pinene (23.5% and 16.8%, respectively) and limonene (23.31% and 5.7%, respectively). Camphor is also present at significant levels (17.3% and 18.6%, respectively) [16, 32, 33].

Also, the essential oils of the dry and fresh leaves of *T. articulata* harvested during the month of December in the region of Marrakech are predominantly by *α*-pinene (23.0%; 41.0%) and bornyl acetate (36.4%; 20.6%) [34].

In addition, in another sample taken in January in the same region, the authors determined bornyl acetate (26.8%) and camphor (22.4%) as the majority compounds, followed by *α*-pinene (7.2%) and borneol (6.4%) [35].

Another study examined the alteration of the majority compounds of essential oils extracted from Barbary thuja leaves from Morocco during shade drying. The majority of compounds are bornyl acetate (30.6%), camphor (18.6%), *α*-pinene (16.8%), limonene (5.7%), and borneol (4.7%). The additional concentration of these five major components increased from 61.1% on the first day to 65.3% on the thirteenth day of shade drying. They also note irregular changes during the drying period. For example, *α*-pinene varies from 23.54% on the first day to 28.78% on the thirteenth day, while bornyl acetate progresses from 30.74% to 22.27% during the same period. The other components remain practically stable during the storage period [29].

As indicated in the literature, these three compounds are the most abundant in the essential oil of the Barbary thuja collected in different regions in Morocco, and the limonene (7.12%), borneol (9.79%), camphene (3.14%), and *α*-thujone (2.51%) are present in significant quantities [27–30]. On the other hand, the majority components of our last work on *Tetraclinis articulata* from Ras Elma Tazakka (mountain, latitude: 34°03′03″; longitude: W 04°10′07″; altitude: 1496 m. The climate was subhumid) and Debdou regions (plain, latitude: 34°03'15”; longitude: W 02°59′15″; altitude: 765 m. The climate was semihumid) showed that the majority components are the bornyl acetate (34.84%; 32.55%), *α*-pinene (11.41%; 18.83%), camphor (11.24%; 11.31%), and limonene (11.94%; 8%) [1].

The quantitative difference in the majority compounds can be explained by the adaptation of the abiotic factors, such as the climate specific to the regions from which the samples come, and geographical factors, such as altitude and soil nature, which orient biosynthesis towards the preferential formation of specific products [36, 37].

### 3.2. Antioxidant Activity

**Figure 3:**
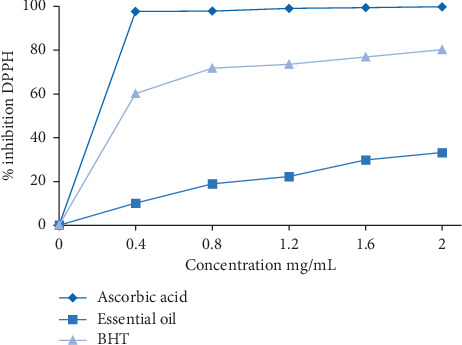
Percentage inhibition curve as a function of the concentration.

The results of the antioxidant activity of the Barbary thuja essential oil are reported in Figure 3.

We found that the essential oils of the aerial part of *Tetraclinis articulata* have a very low antioxidant activity compared with standard antioxidants at a concentration of 2 mg/mL.

According to the literature, the antioxidant activity of *Tetraclinis articulata* has been studied by several authors. The majority of this work confirms our results.

Ben Jemia et al. [38] studied the antioxidant activity of the essential oil of *Thuja* leaves from Tunisia. The results of the DPPH test showed a low activity, with an IC_50_ of the order of 25.50 ± 0.57 *μ*g/mL, two times less important than that of the positive control (BHT) (IC_50_ = 12.0 ± 0.13 *μ*g/mL). It should be noted that bornyl acetate (31.4%), *α*-pinene (24.5%), and camphor (20.3%) are the majority constituents of this essential oil.

Also, El Jemli et al. [35] studied the antioxidant power of the essential oils of the *Thuja* leaves collected in the Marrakech region, and the majority of which are bornyl acetate (26.8%) and camphor (22.4%), followed by the *α*-pinene (7.2%) and borneol (6.4%). These essential oils have low antioxidant activity, with an IC_50_ obtained by the DPPH test, the order of 12.05 × 103 ± 0.24 *μ*g/mL, significantly lower than the standard (IC_50_ = 4.20 ± 0.02 *μ*g/mL).

On the other hand, the essential oil obtained by hydrodistillation, very low in phenolic compound (8.89 mg EAG/g), has the lowest antioxidant activity, with an IC_50_ value of 3681.49 ± 69.33 *µ*g/mL. Polyphenols are known to have a high capacity to entrapment free radicals from the presence of hydroxyl substituents in their aromatic structure [39].

Similarly, the essential oils of leaves, rich in total phenols (320.54 mg EAG/g EO), showed significant antioxidant potential, higher than that of the reference compound butylhydroxytoluene (BHT). The percentage of inhibition obtained by the DPPH test is in the order of 89.3% compared to 50.3% [33].

### 3.3. Antibacterial Activity

The efficiency of essential oil on sensitive microbial strains was determined by the measurement of the minimum inhibitory concentrations (MICs).

The results of the antibacterial activity are given in Table 2.

Teixeira et al. argued that any essential oil with MICs below 2000 *µ*g/mL is considered to have an antimicrobial potential [40].

The results show that the MIC values agree in general with those of the diameters of inhibition, and the essential oils which have induced a large zone of inhibition have the smallest MICs on the strains corresponding.

This is the case with our essential oil, which has proved to be particularly important and effective against *Staphylococcus aureus*, with a very low MIC equal to 1.56 *µ*g/mL, and also for *Pseudomonas aeruginosa* (6.25 *µ*g/mL).

Our results are consistent with those of Lemos et al. [41], who argue that the essential oils of *Rosmarinus officinalis*, which contain between 24.4 and 35.9% of camphor, show significant activity against *S. aureus* with MICs ranging from 0.5 to 2.0 *μ*L/mL.

In addition, the antimicrobial activity of bornyl acetate and camphor has been demonstrated by several works. Indeed, Bougatsos et al. [42], Vagionas et al. [43], and Runyoro et al. [44] report MICs ranging from 1.75 to 4.88 mg/mL for bornyl acetate against several microbial strains. On the other hand, esters and especially bornyl acetate (30.74%) also contribute to the antibacterial effect. Based on the work of Tzakou et al. [45], the essential oils of two chemotypes of *Thymus longicaulis* rich in geranyl acetate for the first and in acetate *α*-terpenyl for the second possess a high antimicrobial activity.

We also found that *Staphylococcus aureus* was the most sensitive to the essential oil. Indeed, most studies report that bacteria in Gram (+) are generally more sensitive than in Gram (−) [46, 47].

Thus, although the antimicrobial activity of essential oil is attributed mainly to its majority compound, the synergistic or antagonistic effect of each of its constituents present in low content is also considered [46, 48, 49].

We can conclude that studies of medicinal plants show a variability in the composition of essential oils that influences the potential of biological activities. This variation was most often correlated with the difference in regions, the harvest period [50–53], environmental and agronomic conditions [54], and the extraction method [55].

## 4. Conclusion

The chemical composition of essential oil leaves harvested from the Benslimane region showed that the majority components are bornyl acetate (35.05%), camphor (11.17%), and *α*-pinene (10.84%). *Tetraclinis articulata* essential oil is very active on bacterial strains, especially *Staphylococcus aureus*, with inhibition zones ranging from 48 to 30 mm diameters. Contrary to the results obtained by the DPPH method, low antioxidant power was found for the essential oil tested compared to the reference compounds.

We can conclude that studies of medicinal plants show variability in the composition of essential oils that affects the potential of biological activities. This variation was most often correlated with the difference in the regions, the harvest period, and environmental conditions.

## Figures and Tables

**Table 1:** Majority essential oil compounds of the aerial part of *Tetraclinis articulata* (Vahl) Masters.

The majority constituents	Percentage (%)
A: *α*-pinene	10.84
B: camphene	3.14
C: *α*-thujone	2.51
D: limonene	7.12
E: camphor	11.17
F: borneol	6.79
J: bornyl acetate	35.05

**Table 2:** Antibacterial activity in the essential oil of *T. articulata* leaves (Vahl).

		E.O
*Staphylococcus aureus* ATCC29213	Ø	48 ± 00
	MIC	1.56 ± 0.00
	MBC	1.56 ± 0.00

*Escherichia coli* ATCC25922	Ø	8 ± 00
	MIC	25 ± 0.00
	MBC	25 ± 0.00

*Pseudomonas aeruginosa* ATCC27853	Ø	30 ± 00
	MIC	6.25 ± 0.00
	MBC	6.25 ± 0.00

MBC: minimum bactericidal concentration; MIC: minimum inhibitory concentration; Ø: zones of inhibition.

## Data Availability

The data used to support the findings of this study are included within the article.
